# Studies of IGF-I and Klotho Protein in Relation to Anabolic-Androgenic Steroid and Growth Hormone Administrations

**DOI:** 10.3389/fspor.2022.829940

**Published:** 2022-03-31

**Authors:** Mikael Lehtihet, Christina Stephanou, Annica Börjesson, Hasanuzzaman Bhuiyan, Anton Pohanka, Lena Ekström

**Affiliations:** ^1^Department of Medicine, Karolinska Institutet, Stockholm, Sweden; ^2^Department of Laboratory Medicine, Karolinska Institutet, Stockholm, Sweden; ^3^Doping Control Laboratory, Department of Clinical Pharmacology, Karolinska University Hospital, Huddinge, Sweden

**Keywords:** doping, anti-doping, IGF-I, klotho, anabolic androgenic steroid, growth hormone

## Abstract

It has been suggested to longitudinally monitor Insulin-like growth factor I (IGF-I) as a biomarker for the detection of recombinant growth hormone (GH). Subsequently, it is of interest to understand any confounders of endogenous IGF-I. Herein we have studied if serum IGF-I concentration is affected by the intake of anabolic androgenic steroids (AAS) and the potential connection between IGF-I and klotho protein. Moreover, the usefulness of klotho as a biomarker for recombinant GH intake was assessed in healthy male volunteers. An ongoing administration of AAS did not affect the levels of IGF-I. Klotho protein was ~30% higher in men with an ongoing AAS use compared to those with previous (>2 months ago) AAS use, and the serum klotho protein correlated negatively with luteinizing hormone (LH) (*r*_s_ = −0.38, *p* = 0.04) and follicle stimulating hormone (FSH) (*r*_s_ = −0.35, *p* = 0.05) levels. Serum IGF-I and klotho concentrations showed no correlation in the AAS using population but showed a strong negative correlation in healthy volunteers (*r*_s_ = −0.86, *p* = 0.006). The intake of recombinant GH did not affect the serum concentrations of the klotho levels. In conclusion, IGF-I was not affected by supra-physiological AAS doses in men. Interestingly, an association between AAS intake and serum klotho was seen. The usefulness of klotho as an androgen biomarker warrants further studies, whereas klotho can be discarded as a promising biomarker for GH doping.

## Introduction

Anabolic-androgenic steroids (AAS) are used among athletes and in society for their muscle building and performance-enhancing effects (Bhasin et al., 1996). In addition to AAS, recreational and elite athletes may co-use recombinant growth hormone (GC; recGH) and insulin-like growth factor I (IGF-I) for the lipolytic and muscle-enhancing effects noted after GH substitution therapy (Chikani and Ho, 2014). To detect recGH doping, the GH2000 score, including serum IGF-I and procollagen type III N-terminal peptide (P-III-NP) biomarkers, is used in World Anti-Doping Agency (WADA) accredited labs. This population-based score can detect high doses of recGH (Guha et al., 2014), whereas detection of lower doses delivers poor results (Lehtihet et al., 2019).

Lately, it has been discussed that IGF-I could be longitudinally monitored, with or without P-III-NP, in an endocrine passport module to increase the true positive rate (Lehtihet et al., 2019; Marchand et al., 2019). Subsequently, it is of interest to understand how the administration of different drugs, including other doping substances, such as AAS, influences the production of IGF-I. In fact, previous studies on exogenous androgen's effect on IGF-I production in healthy individuals are scarce, but indicate that testosterone administration may lead to an increase in IGF-I serum levels (Hobbs et al., 1993; Veldhuis et al., 2005). However, to our knowledge, IGF-I has not been investigated in relation to the supra-physiological doses often used among athletes engaged in e.g., bodybuilding.

Klotho protein has been proposed as a clinical biomarker for GH/IGF status, i.e., in the diagnosis and treatment of acromegaly (Sze et al., 2012; Neidert et al., 2013). It is believed that klotho inhibits insulin and IGF-I pathways (Shahmoon et al., 2014), but the correlation between serum klotho and IGF-I in humans has been inconclusive. These differences may be due to the different populations studied (age, disease status) and the kit used for the IGF-I and klotho analyses (Heijboer et al., 2013; Bidlingmaier et al., 2014). Exogenous recGH therapy has been shown to induce klotho concentrations in some healthy subjects (Adema et al., 2018), indicating that klotho protein could act as a putative marker for GH doping. Moreover, a relationship between klotho and androgens has been suggested as klotho gene promoter includes an androgen receptor element (ARE) and testosterone upregulates the messenger RNA (mRNA) and protein klotho expression in kidney cell lines (Hsu et al., 2014). But to our knowledge, the association of supra-physiological doses of androgens and serum klotho has never been studied.

Here we have studied IGF-I and serum klotho protein in relation to AAS administration in men self-reporting an ongoing or previous AAS use. Moreover, to investigate if serum klotho levels can function as a longitudinal biomarker for GH doping, the klotho protein was studied in relation to GH administration.

## Materials and Methods

### Study Populations

Two cohorts were included in this study: cohort 1 included healthy volunteers administered with recGH and cohort 2 included self-reporting AAS users.

The study population of cohort 1 has been described earlier and included nine healthy male volunteers aged 32–45 years (Lehtihet et al., 2019). The participants were administered with Somatropin (Genotropin^®^, Pfizer Innovations AB, Strangnas, Sweden) daily for 2 weeks (1 or 4 IU/day) with the primary endpoint to study biomarkers of GH doping. This dose regimen mimics doses reported among AAS users (cohort 2) (Borjesson et al., 2020) and athletes (Marchand et al., 2017). The exclusion criteria for participating in the study included cardiovascular diseases, diabetes, hormonal treatment, being under the influence of abused substances (AAS, opioids, cannabis, cocaine, and amphetamine), malignancy within the last 5 years, or being a member of a sports federation. Three blood samples were collected daily for 3 days prior to recGH administration, and two samples were collected during the treatment period: after 7 and 13 days, respectively. Post-samples were taken 1.5, 3, 24, and 48 h after the last injection. All samples were collected in the morning (7.30–10.30). The study was approved by the Ethics Review Board in Stockholm and written informed consent was obtained from all participants before inclusion in the studies.

Cohort 2 consisted of 30 male individuals self-reporting an AAS use within the last year, aged between 20 and 63 years old. They were recruited from our Anti-Doping Hot-Line *via* the snowball sampling approach and the population has been described elsewhere (Borjesson et al., 2020). Participation was commenced after oral and written informed consent and the study was approved by the Ethics Review Board in Stockholm. At inclusion, blood and urine samples were taken, and the weight in kilograms and height in meters were measured to calculate the body mass index (BMI) (kg/m^2^). The participants sat down for 10 min prior to blood sampling, and blood samples were collected from an antecubital vein in serum tubes. Serum was obtained within 4 h by spinning the serum tubes for 10 min at 2,000 g and immediately frozen at −80°C. Testosterone, luteinizing hormone (LH), and follicle-stimulating hormone (FSH) were measured as described and reported previously. The participants were grouped into current AAS users (<2 months) and former AAS users (last AAS intake 2-12 months ago).

### Serum Analyses IGF-I

The IGF-I for the GH administration study was analyzed in our previous report (Lehtihet et al., 2019) and IGF-I analyses of the subjects self-reporting AAS use were quantified with the same WADA accredited method. Briefly, serum IGF-I was measured by a commercially available sandwich-type immunoassay, the Immunotech A15729 IGF-I IRMA (Immunotech SAS, Marseille, France). Six calibrators with different concentrations were used to plot a standard curve. Quality control (QC) samples, QC low and QC high, were used to check the performance of the analysis. Human serum obtained from Sigma Aldrich, ref: H4522, was used as QClow, which gives IGF-1 concentration <200 ng/ml, and QC high was prepared by spiking the same serum with recombinant human IGF-I obtained from Invitrogen, ref: PHG0071, which gives concentration >500 ng/m. A sample volume of 50 μl was applied to the antibody-coated tubes. After the incubation, radioactivity was counted for 5 min with a gamma counter 1282 Compugamma (LKB Wallac). The data were analyzed with spline function curve fitting to determine the concentration of IFG-1 in the samples. To take the age into account, the age-corrected formula calculated from the regression of IGF-I values of healthy adult subjects determined in Hilding et al. (1999) was applied.

### Serum Analyses Klotho

The commercial Human KL(Klotho) ELISA Kit (Catalog Number EKH4368) (Nordic Biosite AB, Täby, Sweden) based on sandwich enzyme-linked immune-sorbent assay technology was used for klotho quantification. To pre-coated anti- klotho antibody wells, standards (7.8–500 pg/ml) and samples of interest were added followed by a biotin-conjugated anti- klotho antibody as detection antibodies. Horseradish peroxidase (HRP)-Streptavidin was added, and unbound conjugates were washed away with wash buffer. Tetramethylbenzidine (TMB) substrates were used to visualize HRP enzymatic reaction. TMB was catalyzed by HRP to produce a blue color product that changed into yellow after adding an acidic stop solution. The O.D. absorbance was read at 450 nm in a microplate reader (SpectraMax^®^ Plus 384 Microplate Reader, Molecular Devices, LLC, San Jose, CA, USA) and the concentration of klotho was calculated from the standard curve (best-fit-purpose) and multiplied by the dilution factor using the SoftMax Pro Software v1.01. (Molecular Devices, LLC, San Jose, CA, USA). All samples were analyzed in duplicates. The kit has been validated regarding recovery (97–102%) and precision (intra-assay CV < 8%, inter-assay CV < 10%) by the manufacturer (Nordic Biosite, Täby, Sweden). The serum volume used in the ELISA reaction was recommended by the kit provider to be empirically tested. Different serum volumes of 1, 10, 50, and 100 ul were tested from two subjects. For the serum results, 75 ul (1:1.33 dilution) was chosen for the serum analyses as this shows linearity and is within the range of the standard curve. For two participants, one with ongoing AAS use and one with previous AAS use, the klotho levels were far above the standard curve and therefore not included in data analyses. Occasional samples from one of the participants in the GH administration study were outside the standard curve and hence this subject was excluded.

### Data Analyses

The statistical analyses were performed using GraphPrism Software version 8 (San Diego, CA, USA). IGF-I and klotho were normally distributed (Shapiro-Wilk test), and the student *t*-test test was used for comparison between ongoing and previous AAS users, whereas the gonadotropins showed non-normal distribution and the Mann-Whitney *U*-test was applied. For the correlation analyses, Spearman rank-order correlation or Pearson's correlation analyses were done depending on the distribution. The coefficient of variation (CV%) was calculated by dividing the SD by the mean value to show intra-individual stability. Differences were considered significant at *p* ≤ 0.05 (2-sided test).

## Results

### AAS Administration and IGF-I

There was no difference in age and BMI between the ongoing and former AAS users, whereas LH and FSH were significantly lower in those with ongoing AAS use (Table 1).

**Table 1:** General characteristics, and hormone values in men with an ongoing AAS (<2 months) and previous AAS (2–12 months).

	**Current AAS users (*n* = 17)**	**Previous AAS users (*n* = 14)**	***P*** **-value**
Age	31.5	33.5	ns
BMI	27.3 + 2.7	29.4 +3.6	ns
LH (IU/LH)	0.10 ± 1.8	5.3 ± 2.6	*p* <0.0001
FSH (IU/L)	0.14 ± 1.5	3.2 ± 3.0	*p* <0.0001
IGF-I (pg/mL)	201 ± 84.1	157 ± 41.7	ns
IGFSD	−0.41 1.5	−1.0 0.9	ns
Klotho (pg/mL)	68.4 ± 20.8	49.3 ± 51.5	*p* = 0.04

*Values presented as median ±SD. For comparison between groups, Whitney U-test or student T-test were applied depending on the distribution*.

*BMI, body mass index; LH, luteinizing hormone; FSH, follicle stimulating hormone; IGF-I, insulin growth factor 1*.

The IGF-I concentrations (range 55–360 ng/mL) were negatively correlated with age (*r* = −0.54, *p* = 0.0006). Therefore, the age-corrected IGFSD values were also applied when comparing IGF-I levels between the groups. There were no significant differences in neither IGF-I and IGFSD between AAS users and former AAS users (Table 1). IGF-I and IGFSD did not correlate with circulatory levels of the gonadotropins (data not shown).

An ongoing use (i.e., within the last two months) of potential interacting substances, such as insulin (*n* = 1), and GH/IGF-I (*n* = 1), displayed IGF-I values of 160 and 279 ng/ml and IGFSD values of −0.30 and −0.59, respectively.

### AAS Administration and Klotho

**Figure 1:**
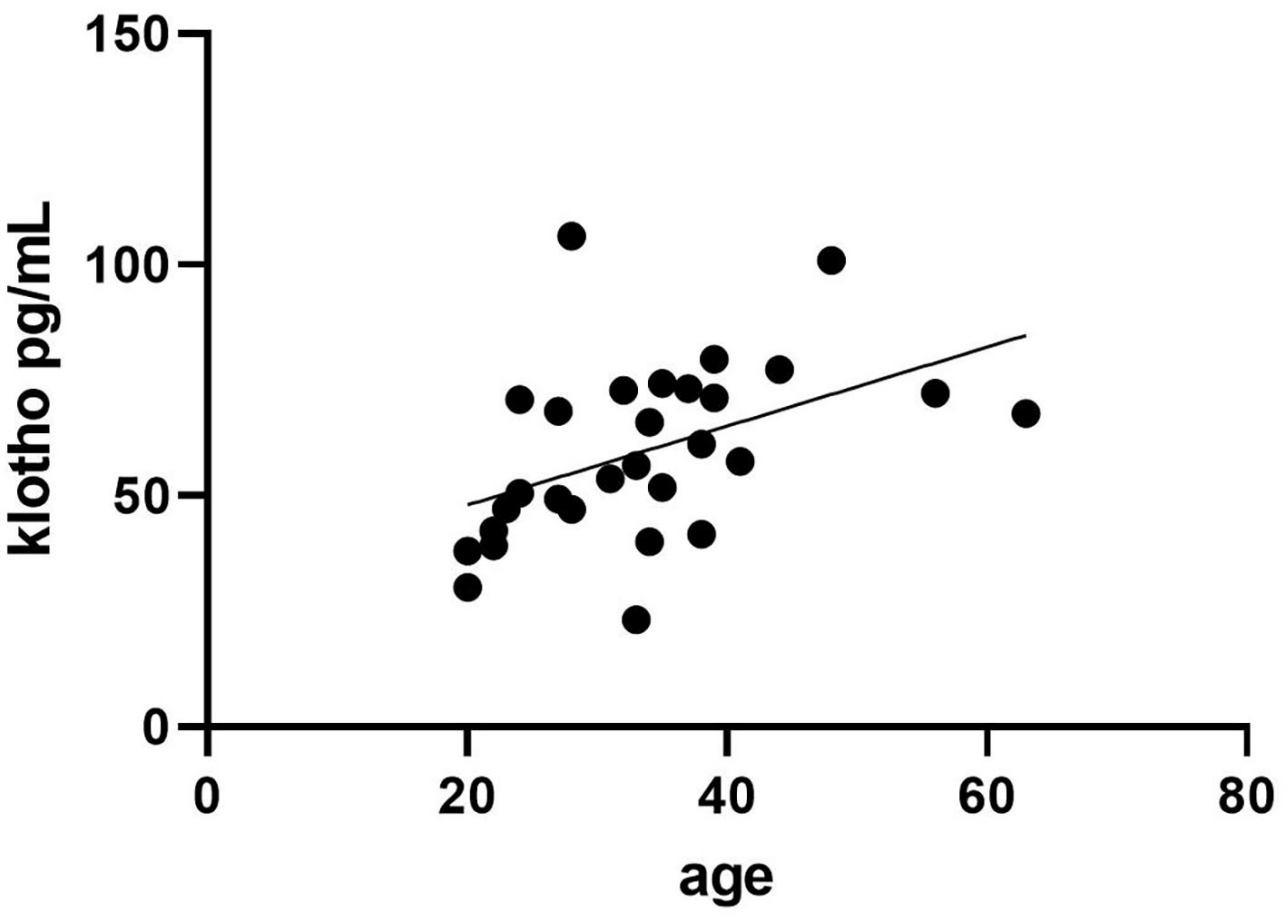
Correlation between age (years) and klotho serum concentrations in 29 male individuals using anabolic androgenic steroids (AAS) within the last year.

A significant correlation between serum klotho levels and age was noticed, increasing klotho concentrations with age, *r* = 0.45, *p* = 0.01, Figure 1.

Klotho serum levels in relation to the time of last AAS intake revealed significantly higher klotho levels in current AAS users compared to those who reported AAS intake longer than 2 months ago, *p* = 0.04, Table 1.

Negative correlations between FSH and LH with klotho serum levels were observed *r*_s_ = −0.353, *p* = 0.05 and *r*_s_ = −0.38, *p* = 0.04.

### GH Administration Study

**Figure 2:**
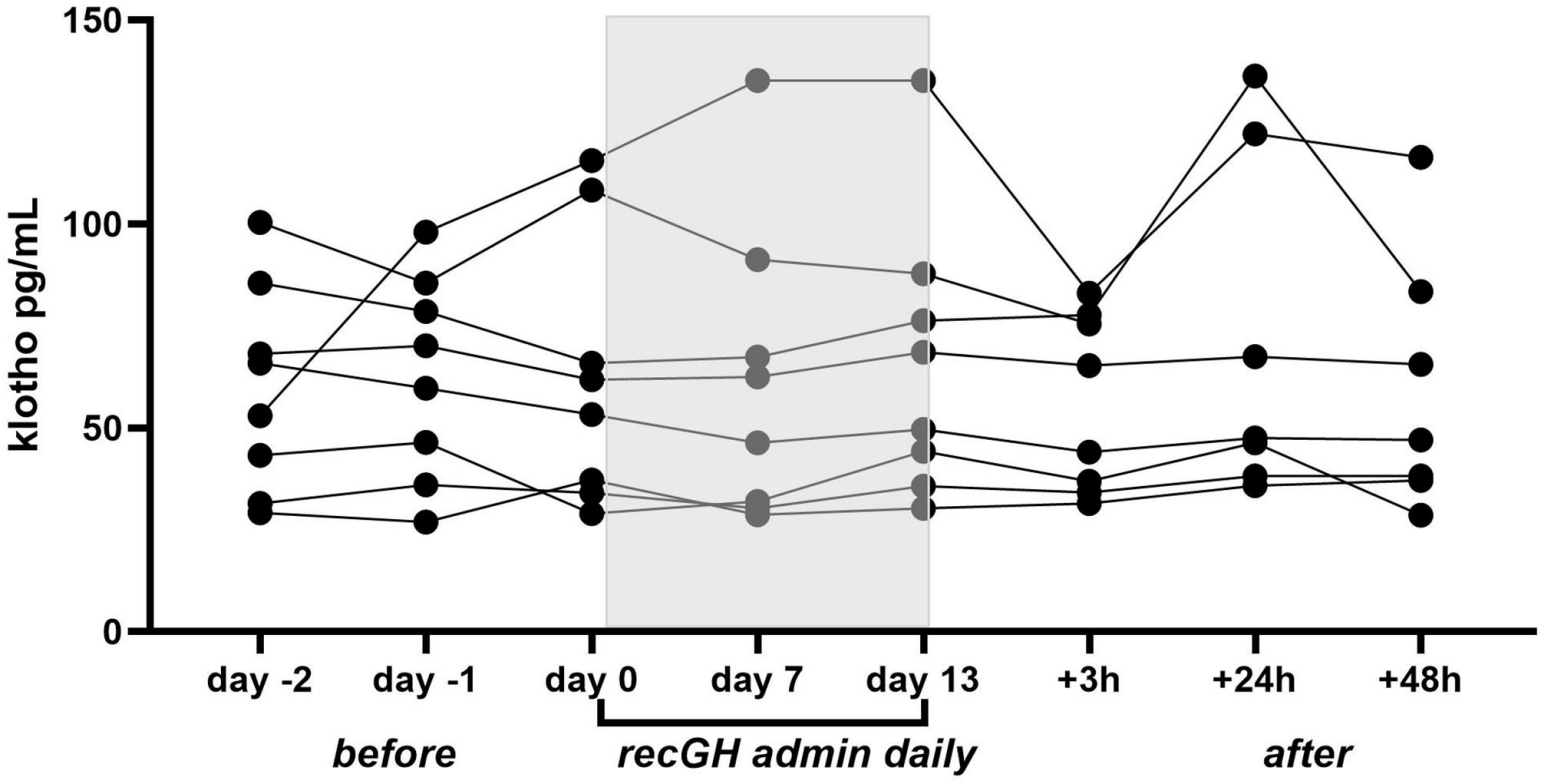
Klotho serum levels in eight participants administered with recGH (0.008–0.051 IU/kg body weight/day) for 2 weeks. Serum samples were analyzed prior to the treatment period (day−2, day−1, and day 0), during treatment (day 7 and 13), and 3, 24, and 48 h post-treatment.

**Figure 3:**
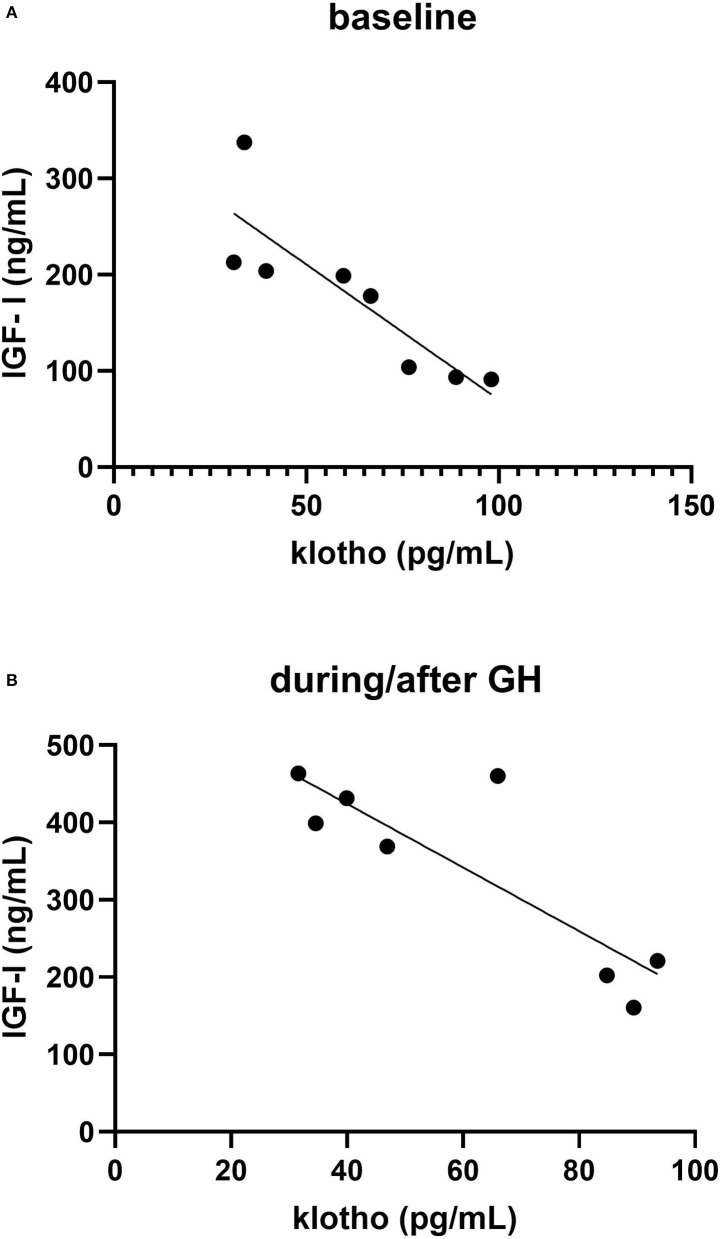
Correlation between IGF-I and klotho in eight healthy volunteers **(A)** mean of three baseline values **(B)** mean of four samples taken during (day 7 and 13) and after (+3 and 24 h) recombinant growth hormone (recGH) administration.

Klotho protein was quantified in serum from eight volunteers from our previous study. Serum samples include samples obtained before recGH administration (*n* = 3/subject) during the treatment period (day 7 and 13) and post GH treatment (+1.5, 3, and 24 h). There were no differences in serum klotho levels between the different time points investigated (*p* = 0.33, Friedman test), Figure 2. The intra-individual variations of the baseline values were lower (range 4.4–26.5% CV) than the inter-individual variation (range 38–59% CV). The klotho levels showed a strong negative correlation to IGF-I concentration before GH administration (*r* = −0.86, *p* = 0.006), Figure 3A. Also, after GH administration, a similar correlation remained (*r* = −0.86, *p* = 0.006), Figure 3B.

## Discussion

This is the first study assessing IGF-I and klotho levels in AAS users. It was shown that the intake of supra-physiological doses of AAS was not associated with circulatory IGF-I levels i.e., there was no difference in IGF-I levels between those with current and former AAS use. Previous studies indicate that androgens increase IGF-I secretion *via* GH (Bondanelli et al., 2003) and it is possible that AAS-induced hypopituitarism abrogates GH responsiveness of androgens. This is in line with clinical studies in men with hypopituitarism where co-administration with GH was needed to obtain an increase in IGF-I after testosterone administration (Gibney et al., 2005).

We did not see any elevated klotho levels in the AAS user with ongoing use of recGH, and to further strengthen the finding that serum klotho is not affected by GH administration, klotho was analyzed in healthy male volunteers that were administered with recGH for 2 weeks. The intra-subject variation in klotho levels was small (4–26 CV%) and the individual values were not perturbed by recGH administration. The result is in line with Adema et al. who did not observe any significant effects on serum klotho on a group level when a similar recGH dose was administered, even if klotho elevation was noted in a few subjects (Adema et al., 2018). So, it is likely that klotho protein cannot function as a valuable biomarker for doping with recGH. Notably, low levels of klotho may also be detected in urine, particularly in patients with GH excess (Schmid et al., 2013) and it is possible that a different response to recGH would have been observed in urine.

A novel finding was that the ongoing use of AAS was associated with higher serum klotho concentrations. Also, the AAS-induced hypogonadism as reflected by LH and FSH repression correlated to klotho levels. Supra-physiological doses of androgens are known to suppress gonadotropins with fast response (Jarow and Lipshultz, 1990). The degree of suppression and the ability to recover after an AAS cycle depends on the duration of AAS use and the cumulative dose (Tan and Scally, 2009) with most being normalized within 6 months, even though in some cases, it may take up to 1 year as seen in our participants (Borjesson et al., 2020) and elsewhere (Sader et al., 2001). It is possible that AAS activates the ARE and influences the expression of the klotho gene resulting in elevated protein levels as has been seen in mice (Hsu et al., 2014). Two AREs have been identified *in silico* in the human klotho promoter region (Hsu et al., 2014) which may explain the higher klotho protein levels in the granulosa cells from women with Polycystic ovary syndrome (PCOS) and hyperandrogenism compared to controls (Mao et al., 2018). Furthermore, a correlation between circulatory T and DHEA and klotho levels in healthy sedentary middle-aged adults has been observed (Dote-Montero et al., 2019). The connection between klotho and androgens in humans warrants further studies.

We noted a strong negative correlation between klotho and IGF-I in the healthy volunteers, whereas in the self-reporting AAS subjects, correlations between klotho and IGF-I/IGFSD could not be discerned. A negative association between klotho and IGF-I is in agreement with the hypotheses that klotho exerts inhibitory effects on the IGF-I pathway as seen in several animal studies (Abramovitz et al., 2011; Shahmoon et al., 2014). A negative association between IGF-I and klotho has been found directly after exercise in healthy subjects (Saghiv et al., 2017). However, in adults with acromegaly positive correlations between IGF-I and klotho have been reported (Sze et al., 2012). Several hypotheses have been proposed for the connection between klotho and the GH/IGF-I pathway, extensively reviewed by Rubinek and Modan-Moses (2016).

The IGF-I and IGFSD values in the AAS users showed large inter-individual variations but were within ranges previously reported in Swedish healthy men (Unden et al., 2002). It is well-known that IGF-I decreases with age and a negative correlation between age and IGF-I were seen among the AAS users. The opposite age pattern was found for klotho, i.e., a strong positive correlation between age and serum klotho concentrations in the AAS users. This is contrary to previous findings where serum klotho levels significantly decreased with age (Koyama et al., 2015). The discrepancy might be that our participants are younger and most of them do not suffer age-associated diseases.

In previous studies it has been observed that athletes display higher IGF-I concentrations than sedentary controls, being highest in athletes engaged in power sports (Eklund et al., 2021). But herein, no differences in IGF-I levels between healthy volunteers and the AAS population were observed. Nor were any differences in serum klotho concentrations between the healthy volunteers and the AAS users noted, but regrettably, both cohorts exhibit lower klotho concentrations that are seen in other studies, including healthy subjects (Dote-Montero et al., 2019). The reason for this may be that the samples have been stored (−80°C) for 3 years and the klotho protein has partly been degraded. However, during method testing, we also included freshly prepared control serum that did show klotho levels in the same range. It is possible that the kit used herein exerts lower sensitivity for klotho than the kits used in other studies, as has been discussed previously (Heijboer et al., 2013). It is possible that only the alternative spliced secreted klotho presents in the circulation (and not the membrane-bound form that has been shredded into the circulation) is detected herein.

There are some limitations with our study that needs to be addressed. Firstly, we only included men in our study populations, and it is possible that gender differences in IGF-I and Klotho response to AAS and GH administration exist. Moreover, regarding the AAS self-reporting population, the timing of the last AAS intake may not be correctly stated. In addition to AAS, several other drugs were used e.g., additional performance-enhancing drugs and drugs taken to eliminate AAS-induced side effects, that might interfere with the results. However, studies of populations self-reporting AAS use is the only way to perform studies of doses often used among bodybuilders, since it is not ethically or medically justified to conduct controlled studies with such doses (60–3,800 mg/week). Another limitation is that the samples were not taken after overnight fasting but rather at different time points to mimic a doping test situation. IFG-I is known to increase after food intake, particularly proteins. Many of the AAS users eat large amounts of proteins as well as dietary supplements which may be confounder herein.

In conclusion, it has been shown that supra-physiological doses of AAS do not affect circulatory IGF-I concentrations. Instead, the AAS exposure was associated with elevated klotho serum levels and the klotho as a biomarker in relation to androgens deserves further investigation. Klotho levels were not influenced by the administration of recGH and suggested not to be a promising biomarker for GH doping.

## Data Availability Statement

The raw data supporting the conclusions of this article will be made available by the authors, without undue reservation.

## Ethics Statement

The studies involving human participants were reviewed and approved by Ethics Review Board, Stockholm. The patients/participants provided their written informed consent to participate in this study.

## Author Contributions

LE and ML were involved in the concept/design of the GH administration study. LE, ML, and AB were involved in the concept/design of the AAS study. LE, HB, and CS performed the quantification of IGF-I and klotho. LE, CS, and AP were responsible for the acquisition of data. LE wrote the first manuscript draft. All authors were involved in the critical revision of the article. All authors listed met the conditions required for full authorship. All authors contributed to the article and approved the submitted version.

## Funding

Grants for performing the rhGH administration study and the collection of samples from AAS users were previously supported by WADA and the Swedish Research Council for Sport Science, respectively, and the analyses herein were supported by the Doping Control Laboratory, Karolinska University Hospital.

## Conflict of Interest

The authors declare that the research was conducted in the absence of any commercial or financial relationships that could be construed as a potential conflict of interest.

## Publisher's Note

All claims expressed in this article are solely those of the authors and do not necessarily represent those of their affiliated organizations, or those of the publisher, the editors and the reviewers. Any product that may be evaluated in this article, or claim that may be made by its manufacturer, is not guaranteed or endorsed by the publisher.
